# Misreporting of Energy Intake From Food Records Completed by Adolescents: Associations With Sex, Body Image, Nutrient, and Food Group Intake

**DOI:** 10.3389/fnut.2021.749007

**Published:** 2021-12-13

**Authors:** Louise Jones, Andy Ness, Pauline Emmett

**Affiliations:** ^1^Centre for Child Academic Health, Bristol Medical School, University of Bristol, Bristol, United Kingdom; ^2^Bristol Dental School, University of Bristol, Bristol, United Kingdom

**Keywords:** adolescence, misreporting, energy intake, body composition, ALSPAC, dieting, body image, food groups

## Abstract

**Background and Objectives:** A healthy diet during adolescence is important for growth and pubertal development. Assessing the diet of adolescents may be challenging as the behavioural factors and food habits which impact on what they eat may also affect how they report dietary intake. This study assesses factors associated with the misreporting of dietary intake.

**Methods:** Adolescents (*n* = 4,844; average age 13.8 years) from the Avon Longitudinal Study of Parents and Children (ALSPAC) completed a 3-day diet record. Misreporting was estimated using an individualised method, and adolescents were categorised by reporting status. Foods were categorised as core and noncore foods to evaluate diet quality. Body composition measurements were recorded at a research clinic. Information on dieting, weight concern, family socioeconomic status, and parental BMI were collected *via* questionnaires. Binary logistic regression was performed, in boys and girls separately, to investigate factors associated with underreporting of dietary intake.

**Results:** Girls were much more likely than boys to be dissatisfied with their weight and to diet, but showed similar levels of underreporting (~67%). In adjusted regression analysis underreporters (UR) were more likely to be overweight or obese: OR in boys 2.8 (95% CI 1.7–4.8) and in girls 2.2 (95% CI 1.5–3.2). Dissatisfaction with weight and dieting were positively associated, and perception of being underweight negatively associated with underreporting in boys. Perception of being overweight, dieting, and exact age were positively associated with underreporting in girls. UR obtained a greater percentage of energy from protein and a smaller percentage of energy from fat; they reported greater intake of core foods and lower intakes of non-core foods than plausible reporters.

**Conclusion:** A large proportion of adolescents underreported their dietary energy intake. This was associated with their body weight status and body image and had a differential effect on their estimated food and macronutrient intakes. Assessment of misreporting status is essential when collecting and interpreting dietary information from adolescents.

## Introduction

Adolescence is a key developmental stage in the lifecycle. It is a transitional period between childhood and adulthood and is characterised by rapid physical growth and pubertal development alongside behavioural and emotional changes. From a nutritional point of view, it is a crucial period as total nutrient requirements are greater than at any other age and hence the need to provide for optimum growth and pubertal development (1, 2).

Behavioural changes that occur in adolescence may have a negative impact on nutritional status. Parental control of diet reduces as adolescents take greater control regarding food choices and eating outside the home becomes more frequent. Many choose to snack between meals, skip meals, and consume greater amounts of foods rich in fat and/or sugar, and low in essential nutrients (3, 4). Adolescent girls, in particular, may start to diet, as they become increasingly aware of body image. These weight reduction diets could be unhealthy and cause nutrient deficiencies. An unhealthy diet during this period may increase the risk of overweight and obesity. Excess weight in adolescence may track into adulthood and increase risk of chronic diseases such as diabetes and heart disease later in adulthood (5).

Therefore, it is of great relevance to public health to assess the nutritional intake of adolescents, but accurately assessing the diet of adolescents may be challenging as the behavioural factors and food habits which impact on what they eat may also impact on their ability and willingness to accurately report dietary intake. There is limited research on the issue of misreporting of energy in adolescents compared with adults. In a literature review conducted by Forrestal., the prevalence of misreporting in children and adolescents varied greatly with a range of 2–85% for UR 3–46% for overreporters (OR) (6). Several studies consistently reported that higher weight, BMI, and adiposity were associated with underreporting in adolescents as for adults (7, 8). There is less consensus regarding other variables potentially associated with misreporting such as sex, sociodemographic status, weight concern, and parental characteristics. Very few studies have collected all these variables to ascertain which factors are the most important predictors of misreporting.

This study uses a large British cohort of 13-year-olds to assess factors associated with the misreporting of dietary intake. It investigates associations with body image and socio-economic background and the impact of misreporting on dietary intakes keeping the sexes separate.

## Methods

### Subjects

The participants were part of the Avon Longitudinal Study of Parents and Children (ALSPAC), a geographically based prospective cohort study investigating the factors influencing the health, growth, and development of children. Pregnant women residing in Avon, UK with expected dates of delivery between 1 April 1991 and 31 December 1992 were invited to take part in the study; 14,541 women enrolled. Seven years after these dates additional children from eligible pregnancies were recruited. This provided a baseline group of 15,454 pregnancies, resulting in 15,589 foetuses. Of these 14,901 infants were alive at 1 year of age. The aims and study design have been described in more detail elsewhere (9, 10). The study website contains details of all the data that is available through a fully searchable data dictionary and variable search tool http://www.bristol.ac.uk/alspac/researchers/our-data/. Informed consent for the use of data collected *via* questionnaires and clinics was obtained from participants following the recommendations of the ALSPAC Ethics and Law Committee at the time.

### Dietary Assessment

Dietary data were collected from 2004 to 2006 when the adolescents (mean age 13.8 years) and their main carer were invited to attend a research clinic. Before the visit, the adolescents were instructed to record in a structured 3-day diet diary all the food and drinks consumed (using household measures), for 1 weekend day and 2 weekdays (self-selected and not necessarily consecutive). A short questionnaire accompanied the diary asking detailed questions about foods and drinks commonly consumed. It covered the types and thickness of slices of bread and fat spread used, details of drinks such as milk, tea, coffee, and soft drinks, and also included recording the volumes of cups/mugs/flasks usually used. It was used to improve the accuracy of the dietary data collection. At the clinic, a nutrition fieldworker reviewed the diary and interviewed the adolescent and carer to gain further information on portion size, cooking methods, and any food/drink missed out. If no diary had been brought to the clinic, the fieldworker conducted a 24-h recall for the previous day. There are some participants who completed both a 24-h recall and a 3-day diet diary. In this analysis participants with data for only 1 day were excluded.

The completed diaries were coded by the same fieldworker using the computer program DIDO (Diet In, Data Out). This program was designed for direct entry of food records (11); it is based around a hierarchical menu of food names with portion weights. An advantage of the program is that new foods can be added, and portion sizes can be modified for different age groups. When information on portion size was missing from the diary, average portion sizes for similar aged adolescents derived from a weighed dietary intake survey were used (12, 13). Alternatively, portion sizes were informed based on the manufacturer's information or by reference to adult portion sizes.

The databank used for nutrient analysis included the fifth edition of McCance and Widdowson's food tables and supplements (14–22). Additional up-to-date nutrient information was obtained from the National Diet and Nutrition Survey (NDNS) database (12) and manufacturers. The coded diaries were checked against the originals by a different fieldworker and any errors identified were corrected. Diaries that produced very high or low estimates for key nutrients (top 5%) were rechecked. Free sugars were calculated according to the definition in Dietary Reference Values (DRV) (23). The average weight of food groups consumed based on those used in NDNS were calculated. Nutrients from dietary supplements were not included in the analysis.

### Core, Non-core Foods, and Soft Drinks

To assess differences in reporting of food intakes in a meaningful way, foods were divided into core and non-core foods as defined by the Australian Guide to Healthy Eating (24). The Australian Guide to health eating is very similar to guidelines for the UK and has been used in other studies of UK adolescents (25). Core foods are nutrient-rich foods such as meats, fruits, and vegetables whereas non-core foods tend to be energy-dense and nutrient-poor, and include processed foods with added fat and/or sugar. Intake of regular soft drinks, diet soft drinks, and fruit juice were also assessed. To facilitate direct comparisons between particular food groups consumed by UR and plausible reporters (PR), the weights of foods and drinks per unit energy (MJ) were calculated.

### Misreporting Classification

The probable levels of misreporting were investigated using an individualised method by comparing the ratio of reported energy intake (EI) with total energy expenditure (TEE) (26). TEE was calculated for each child based on their body weight, using separate equations for boys and girls, with an increment added for energy used in growth (27).

Boys: 1.298 + (0.265 x weight) – (0.0011 x weight x weight)
Girls: 1.102 + (0.273 x weight) – (0.0019 x weight x weight)

The validity of reported energy intake was assessed by comparing the calculated TEE with EI. The coefficient of variation of daily energy intake was calculated, and this accounts for intraindividual variation in reported energy intake, over the number of days of assessment. The coefficient variation was calculated separately for boys and girls; it was 19.0% for boys and 20.2% for girls. Adolescents were classified as UR, PR, or over-reporters (OR) according to these cut-off values. Physical activity was not assessed during the dietary recording period, and so no adjustment was made for individual activity level, although an allowance had been made for moderate activity in the equation used for misreporting (27).

### Body Composition Measurements

At the 13-year clinic, the height of the participants was measured to the last complete millimetre using a Harpenden stadiometer and weight was measured to the nearest 50 g using a Tanita Body Fat Analyser (model TBF 401A). Whole body Dual Energy x ray absorptiometry (DXA) scans using a Prodigy scanner (Lunar Radiation Corp, Madison, WI, USA) were carried out to derive total fat mass (FM) and fat-free mass (FFM). BMI was calculated using the standard equation: BMI = weight (kg)/ [height (m)]^2^. Overweight and obesity were defined using age- and sex-specific cut-off points identified by Cole et al. using 1,990 reference centiles, with underweight defined as in Cole et al. (28).

### Dieting and Weight Concern

In a questionnaire sent at 13 years of age, the adolescent was asked “how satisfied are you at the moment with your weight”, responses were recoded into two categories satisfied and dissatisfied. The participants were also asked to describe their weight, the responses were recoded as underweight, right weight, or overweight. A further question asked, “during the past year, did you go on a diet to lose weight or keep from gaining weight?”, the responses were recoded into ever dieted or never dieted.

### Socio-Economic Status, Maternal Educational Level, and Parental Body Composition

A self-reported questionnaire at 32 weeks gestation was used to collect data on the educational level of the mother and occupation of the father. Educational status was grouped as: low [no qualification, CSE (Certificate of Secondary Education; national school exams at age 16-years), or vocational qualifications], medium [O-Levels (national school exams at 16-years, higher than CSE)], or high [A-Levels (national qualifications at 18-years), degrees or postgraduate qualifications]. Paternal social class based on occupation was grouped as low (skilled manual, partly skilled, and unskilled) and high (professional, managerial, and skilled non-manual). At 12 years after the child's birth the mothers were asked how much take-home income the family had each week, the responses were recoded as < £240, £240–£429, and £430 or more. Parents self-reported their heights and weights pre-pregnancy, and parental BMI was calculated and classified as under/healthy weight or overweight/obese.

### Statistical Methods

The statistical software package SPSS version 23 (SPSS Inc., Chicago, IL, USA) was used to perform the analyses. Characteristics of the study sample stratified by sex and reporting accuracy are presented as means for continuous variables and as percentages for categorical variables. χ^2^ Tests and *t-*test were used to compare frequencies and means respectively, and the threshold for statistical significance was set at *p* ≤ 0.05. A binary logistic regression using the backward stepwise method (using the likelihood ratio) was performed to investigate the factors associated with underreporting as the outcome variable. In the regression analysis weight status based on BMI was used to represent the continuous variables weight, height, and BMI which were highly correlated.

## Results

### Response Rates

A total of 11,088 children were invited to attend the clinic for assessment at 13 years (mean age 13.8 years), of which 6,136 attended (54.2%). Diet diaries for two or more days were available for 4,844 (78.9% of attendees); of these, 205 provided 4 days of intake (24 h recall + 3 days), 4,141 provided 3 days and 498 provided 2 days. Participants who attended the clinic and those who did not were compared for baseline characteristics (data not shown). Boys were less likely than girls to attend the clinic (*p* = 0.001). Mothers of adolescents who did not attend the clinic were younger (*p* < 0.001), had lower educational qualifications (*p* < 0.001), and a greater prepregnancy BMI (*p* = 0.006) than mothers of attendees. Paternal social class was also lower (*p* < 0.001).

### Energy Intake Misreporting

A large proportion of the cohort was identified as underreporting (67.2%). There were very few OR (1.5%) so these were excluded from further analysis. The number of under reporters and PR by sex and weight status are presented in Table 1. The amount of underreporting was similar for boys (67.8%) and girls (66.6%). Overweight/obese adolescents were disproportionately more likely to be UR (87.1%) than PR (12.9%).

**Table 1:** Differences by sex and weight status in 13-year-old adolescents grouped by their accuracy of reporting of energy intake in food records.

	**Under-reporters^a^**		**Plausible reporters^a^**		**Total**
	**n**	**%**	**n**	**%**	
All participants	3,254	67.2	1590	32.8	4,844
Sex					
Boys	1,576	67.8	747	32.2	2,323
Girls	1,678	66.6	843	33.4	2,521
Weight status^b^					
Under/healthy weight	2,305	61.4	1,449	38.6	3,754
Overweight/obese	949	87.1	141	12.9	1,090

a *Using Torun (27)*.

b *BMI categorised using cut-offs by Cole et al. (28)*.

### Characteristics of the Participants

Participant characteristics are presented in Table 2 stratified by reporting group and sex. Compared with PR, adolescents of both sexes in the underreporting category had higher mean weight, height, BMI, FM, TEE, and percentage of energy from protein. They were also slightly older and had lower intakes of energy from fat. Their measured weight status showed that the level of overweight/obesity in the UR was very similar in boys and girls (~29%) although the level of overweight/obesity in PR was higher in girls (11.6%) than boys (5.7%). UR, both boys and girls, were more likely to be dissatisfied with their current weight and more likely to perceive themselves as overweight compared with PR. However, a greater proportion of girls, than boys, in both reporting groups stated that they were dissatisfied with their current weight and considered themselves overweight. UR were more likely to have dieted in the past year compared with PR, and again a much greater proportion of girls reported dieting compared with boys, regardless of misreporting status. In these measures of body image, taken as a whole, girls were more out of step with their actual weight status than boys. UR were more likely to have parents who were classified as overweight/obese than PR. The only socio-demographic variable associated with reporting status was maternal education and only among girls.

**Table 2:** Descriptive characteristics of 13-year-old adolescents by sex and dietary energy reporting group.

	**Boys**					**Girls**				
	**Under-reporters** **(*****n*** **= 1,576)**		**Plausible reporters** **(*****n*** **= 747)**			**Under-reporters** **(*****n*** **= 1,678)**		**Plausible reporters** **(*****n*** **= 843)**		
	**Mean**	**sd**	**Mean**	**Sd**	P^*^	**Mean**	**Sd**	**Mean**	**sd**	P^*^
Age (months)	165.9	2.3	165.7	2.3	0.030	166	2.3	165.8	2.5	0.05
Weight (kg)	56.7	12.0	49.8	9.7	<0.001	56.1	10.3	51.1	9.8	<0.001
Height (cm)	165.3	8.4	163.8	9.1	<0.001	162.2	6.1	161.5	6.8	0.007
BMI (kg/m^2^)	20.7	3.5	18.4	2.2	<0.001	21.3	3.5	19.5	3.1	<0.001
Fat mass (kg)	12.7	8.2	7.5	4.2	<0.001	17.7	7.5	13.7	6.9	<0.001
Lean mass (kg)	40.0	6.9	41.1	7.4	<0.001	35.4	3.9	34.7	4.2	<0.001
EI (mJ/d)	7.96	1.47	10.83	1.48	<0.001	6.52	1.1	9.12	1.0	<0.001
TEE (mJ/d)	12.6	1.56	11.67	1.41	<0.001	10.23	0.5	9.91	0.6	<0.001
Protein (% of total EI)	15.1	2.7	13.9	2.3	<0.001	14.9	3.0	13.7	2.3	<0.001
Fat (% of total EI)	34.3	5.3	35.4	4.9	<0.001	34.5	5.5	36.0	4.8	<0.001
Carbohydrate (% of total EI)	50.7	5.6	50.7	5.2	0.740	50.7	5.8	50.4	5.2	0.133
	n	%	n	%		n	%	n	%	
Weight status										
Under healthy weight	1,126	71.5	704	94.3	<0.001	1,179	70.3	745	88.4	<0.001
Overweight obese	450	28.5	43	5.7		499	29.7	98	11.6	
Adolescents body weight satisfaction										
Satisfied	772	77.6	488	92.2	<0.001	705	58.4	469	76.5	<0.001
Dissatisfied	223	22.4	41	7.8		502	41.6	144	23.5	
Adolescents perception of weight										
Underweight	161	13.8	153	25.8	<0.001	115	8.3	121	16.9	
Right weight	689	59.2	387	65.3		766	55.1	464	65.0	<0.001
Overweight	313	26.9	53	8.9		508	36.6	129	18.1	
Reported dieting in last year										
Ever went on diet	187	16.0	19	3.2	<0.001	611	43.9	159	22.1	<0.001
Never went on diet	979	84.0	571	96.8		780	56.1	560	77.9	
Maternal educational status										
Lower education	280	19.3	141	20.3	0.880	302	19.9	125	15.9	
Lower secondary education	510	35.2	243	34.9		546	35.9	271	34.6	0.021
High secondary/University	658	45.4	312	44.8		672	44.2	388	49.5	
Paternal Social class										
Low	491	36.5	223	34.8	0.463	536	37.1	263	35.8	
High	855	63.5	418	65.2		908	62.9	472	64.2	0.540
Weekly Family income										
< £240	121	10.1	52	8.9	0.691	136	10.7	58	9.1	0.506
£240 - £429	344	28.7	174	29.7		361	28.3	190	297	
£430 or more	734	61.2	359	61.4		777	61.0	391	61.2	
Maternal BMI status										
Under/healthy weight	1,183	80.9	595	84.8	0.027	1,229	79.7	679	86.1	<0.001
Overweight/obese	280	19.1	107	15.2		313	20.3	110	13.9	
Paternal BMI status					<0.001					
Under/healthy weight	555	50.6	331	62.6		584	50.6	382	62.9	<0.001
Overweight/obese	542	49.4	198	37.4		570	49.4	225	37.1	

* *χ^2^ tests and t test were used to compare frequencies and means*.

### Associations Between Underreporting and Explanatory Variables

The results of the binary logistic regression analysis are presented in Table 3 stratified by sex. Overall UR regardless of sex were more likely to be overweight or obese: OR in boys 2.8 (95% CI 1.7–4.8) and in girls 2.2 (95% CI 1.5–3.2). In boys dissatisfaction with weight and dieting were positively associated with underreporting, whereas the perception of being underweight was negatively associated. However, in girls there was no association with weight dissatisfaction but perception of being overweight and dieting were positively associated with underreporting. Among girls only there was a positive association between fathers being overweight/obese and underreporting, but mother's education level and weight status did not survive adjustment for the other variables. Diets of UR were characterised by a greater contribution of protein to energy intake and a smaller contribution of fat to energy intake compared with those of PR; this was true for both boys and girls.

**Table 3:** Binary logistic regression: associations of age, weight status, body image, dieting, and sociodemographic variables with underreporting of dietary energy intake (odds ratio and 95% confidence intervals) in 13-year-old adolescents.

	**Boys^a–c^**				**Girls^d–f^**			
	**Step 1**		**Final step**		**Step 1**		**Final step**	
	**OR**	**95% CI**	**OR**	**95% CI**	**OR**	**95% CI**	**OR**	**95% CI**
Age (months)	1.05	0.99, 1.12			1.07	1.0, 1.13	1.07	1.0, 1.13
*P*	0.105				0.014		0.017	
Weight Status							
Under/healthy weight	1		1		1		1	
Overweight/obese	2.9	1.7, 5.0	2.8	1.7, 4.8	2.0	1.3, 2.9	2.2	1.5, 3.2
*P*	<0.001		<0.001		0.01		<0.001	
Adolescents body weight satisfaction								
Satisfied	1		1		1			
Dissatisfied	2.1	1.3, 3.6	2.2	1.3, 3.6	1.2	0.85, 1.7		
*P*	0.004		0.003		0.293			
Adolescents perception of weight								
Underweight	0.6	0.4, 0.8	0.6	0.4, 0.8	0.72	0.5, 1.0	0.7	0.5, 1.0
Right weight	1		1		1		1	
Overweight	0.9	0.5, 1.5	0.9	0.5, 1.5	1.5	1.0, 2.1	1.6	1.1, 2.2
*P (trend)*	0.06		0.002		0.022		0.001	
Reported dieting in last year								
Yes went on diet	3.2	1.7, 6.2	2.7	1.4, 5.1	1.7	1.3, 2.3	1.8	1.3, 2.4
Never went on diet	1		1		1		1	
*P*	<0.001		0.003		0.001		<0.001	
Maternal educational status	NS		NS					
Lower education					1			
Lower secondary education					0.98	0.67, 1.45		
High secondary/University					0.81	0.55, 1.2		
*P (trend)*					0.294			
Maternal BMI status								
Under/healthy weight	1					1		
Overweight/obese	0.9	0.6, 1.3			1.25	0.86, 1.8		
*P*	0.612				0.244			
Paternal BMI status								
Under/healthy weight	1				1		1	
Overweight/obese	1.0	0.8, 1.3			1.3	1.0, 1.7	1.4	1.1, 1.7
*P*	0.865				0.028		0.020	
Protein (% of total EI)	1.2	1.1, 1.3	1.2	1.1, 1.3	1.16	1.1, 1.2	1.16	1.1, 1.22
*P*	<0.001		<0.001		<0.001		<0.001	
Fat (% of total EI)	0.97	0.94, 1.0	0.97	0.95, 1.0	0.95	0.93, 0.97	0.95	0.93, 0.97
*P*	0.024			0.024	<0.001		<0.001	

a *Variables removed on step 2 paternal weight status*;

b *variables removed on step 3 maternal BMI status*;

c *variables removed on step 4 age of study child*.

d *variables removed on step 2 maternal education*;

e *variables removed on step 3 participants satisfaction with weight*;

f *variables removed on step 4 maternal BMI status*.

### Core Foods and Non-core Foods

Table 4 shows the energy-adjusted core and non-core food intakes reported by the UR compared with PR stratified by sex. Underreporting boys compared with plausible reporting boys reported greater intakes of most core foods except for “milk, cheese, and yoghurt” and fruit. Underreporting girls reported greater intakes of most core foods except breakfast cereals, where intakes were similar, and “milk, yoghurt, and cheese” which was higher in PR. UR, regardless of sex, had greater intakes of total core foods than PR.

**Table 4:** Mean weight of energy adjusted intakes of core foods, non-core foods (g/MJ) and drinks (ml/MJ) for under reporters compared to plausible reporters, stratified by sex.

**Foods**	**Boys**			**Girls**		
	**Under-reporters** **(*n* = 1,576)**	**Plausible** **(*n* = 747)**	***p*-value^a^**	**Under-reporters** **(*n* = 1,678)**	**Plausible** **(*n* = 843)**	***p*-value^a^**
**Core Foods**						
Breakfast cereals (g/mj)	4.3	3.9	0.010	3.4	3.2	0.108
Bread – all types (g/mj)	11.4	10.5	0.001	11.3	10.1	<0.001
Pasta and Rice (g/mj)	12.4	10.1	<0.001	13.8	11.5	<0.001
All vegetables (g/mj)	9.4	7.4	<0.001	11.9	9.4	<0.001
Plain potatoes (g/mj)	5.0	4.3	0.015	5.7	4.5	<0.001
All fruit (g/mj)	7.9	8.8	0.052	13.1	10.7	<0.001
Milk, cheese & yoghurt (g/mj)	33.6	34.2	0.570	27.8	30.4	0.011
Beef, lamb & pork(g/mj)	5.7	4.7	0.001	5.2	4.3	0.001
Chicken dishes (g/mj)	4.3	3.5	<0.001	6.3	4.7	<0.001
Oily fish (g/mj)	0.8	0.6	0.011	2.9	1.9	0.004
Total core foods (g/mj)	94.8	88.0	<0.001	101.4	90.7	<0.001
**Non-core foods**						
Coated poultry (g/mj)	1.0	0.8	0.020	1.0	0.9	0.277
Burgers and sausages (g/mj)	2.2	1.9	0.037	1.7	1.4	0.077
Potatoes with fat (g/mj)	7.7	6.3	<0.001	7.2	6.4	0.008
Biscuits, cakes & buns (g/mj)	4.5	6.4	<0.001	4.9	6.4	<0.001
Puddings and ice cream (g/mj)	2.9	3.9	<0.001	3.2	4.0	0.001
Savoury snacks (g/mj)	1.7	1.8	0.318	1.9	1.8	0.230
Chocolate confectionery (g/mj)	1.8	2.3	<0.001	1.8	2.3	<0.001
Sugar confectionery (g/mj)	0.7	0.9	0.072	0.9	1.1	0.104
Total non-core foods (g/mj)	22.6	24.3	0.001	22.6	24.3	<0.001
Full sugar soft drinks (ml/mj)	20.7	23.4	0.035	16.6	17.3	0.535
Diet soft drinks (ml/mj)	50.8	36.6	<0.001	33.3	23.1	<0.001
Fruit Juice (ml/mj)	18.3	19.1	0.426	21.1	21.4	0.797

a *<0.05*.

There were similarities between the diets of UR regardless of sex for the amounts of non-core foods eaten. They reported higher intakes of coated poultry and fried potatoes, but lower intakes of snack foods such as biscuits, cakes and buns, and chocolate confectionery than PR. Overall, it was that reported noncore food intake was lower in UR. In both sexes, UR reported higher intakes of diet soft drinks compared with PR.

## Discussion

In this work, we investigated a range of factors which may predict the underreporting of dietary intake in a group of British adolescents, with particular reference to differences between boys and girls. Adolescents were categorised as either UR or PR of energy intake using standard equations. As expected, UR in both sexes had greater adiposity, and being overweight/obese was strongly associated with underreporting. This was confirmed by DXA as being due to higher FM rather than lean mass. Girls were much more likely than boys to be dissatisfied with their weight, perceive themselves as overweight, and have dieted in the past year despite their measured levels of overweight/obesity being fairly similar. Dieting in girls was more prevalent than their measured level of overweight/obesity could warrant in both UR and PR. Being dissatisfied with their weight was positively associated with underreporting in boys but not girls, whereas perception that they were overweight was associated in girls but not boys. Dieting was associated with underreporting in both sexes. In both sexes' UR reported lower intakes, on average, of dietary fat (energy%) and of non-core food items high in fat and sugar, and higher intakes of protein (energy%) and of core food items than PR.

There was a high level of underreporting of energy intakes in this work (67.2%). Percentages of underreporting are not always directly comparable with other studies as a variety of factors can explain the variability observed, such as the method of dietary assessment, number of days recorded, the equations used to calculate misreporting, the cut-off values applied, the age of participants, and the availability of measured physical activity (4). The best method for measuring total energy expenditure is the doubly labelled water method, but the drawbacks are its expense and the need for extensive resources making it unsuitable for use in large studies. In this study, we used the equations of Torun to estimate levels of misreporting; these take sex and body weight into consideration but includes only a standard increment for moderate physical activity. Although the proportion of UR identified in this study was high, it is comparable with another study of British adolescents aged 11–18 years (73%) using a similar dietary assessment method (29). A review of the literature covering misreporting in children and adolescents reported that the prevalence of underreporting ranged from 2–85% (6). The levels of overreporting were very low and so were not investigated further.

This work did not show a difference in the rates of underreporting between adolescent boys and girls, this is similar to several studies (30, 31). In this regard, adolescents differ from adults where women are more likely to underreport than men (32).

Several studies investigating children with a range of ages have found an inverse relationship between age and level of underreporting (26, 33). In this cohort at age 10 years, 36% of the participants were classified as UR (34), considerably fewer than at 13 years. In the current work, although the participants age range is narrow, the UR, regardless of sex, were shown to be slightly older, for the age of the girl remained in the final step of the regression model. There could be many reasons why underreporting increases with age. When children are young, they are more likely to have parental assistance completing food records. The eating patterns of adolescents may change as they take greater autonomy regarding food choices; snacking may increase especially outside the home and is more likely to be forgotten (4). Children moving into adolescence may be less motivated to complete the foods records, which are time-consuming, thus leading to reporting errors (4).

Approximately one fifth of the adolescents in this work classified as overweight/obese based on BMI cut offs using international standards, and the majority of these were categorised as UR (87%). UR in both sexes were taller, heavier, and had greater FM than PR, consistent with findings of other works (30, 35). Fat and lean mass in adolescents increase at different rates depending on pubertal status, making it harder to assess adiposity using BMI (36). Most studies rely on BMI, which does not differentiate between fat and lean mass, to assess adiposity. This study has an advantage in that it directly measured adiposity using DXA, so it could confirm that UR, regardless of sex, had greater FM but similar lean mass compared with PR. It could be hypothesised that similar to adults who underreport are more likely to be overweight/obese, the children of obese/overweight parents will also under-report their dietary intakes. In the univariate analysis there was a strong association between overweight/obesity in both parents and underreporting in both boys and girls. However, in the final step of the regression there was no association with parental overweight/obesity in boys, but in girls there remained an association with paternal weight status. It might have been expected that teenage girls would be more influenced by their mothers. Lanctot et al. found a similar association that girls classified as UR had parents with higher BMIs (35).

The finding that overweight/obese adolescents were more likely to underreport energy intake than their normal/underweight peers may be due to an unconscious or subconscious bias in misreporting intakes of snacks or food items often considered to be unhealthy. However, it is possible that UR were dieting on the recording days, and so they truthfully reported low food and energy intakes. This possibility is supported by our finding that dieting practise was positively associated with underreporting. Almost half of the girls and 16% of the boys classified as UR stated they had been on a diet in the last year. Another possibility is that adolescents, particularly girls, are aware of body image and that this affects the way they report their diet. The adjusted regression models showed that boys who were dissatisfied with their body weight and girls who perceived themselves as overweight were more likely to underreport energy intake. It was very striking from these results that girl's perception of being overweight, and their frequency of dieting far exceeded their measured level of overweight/obesity (37, 38). Misperception of weight status and inappropriate dieting were much more prevalent in girls than boys.

Underreporting was also associated with the macronutrient composition of the diet, with UR having a greater energy contribution from protein and a lower energy contribution from fat than PR. There was no difference in the contribution to energy from carbohydrate. Similar findings have been observed in studies of both adolescents and adults (39). The differences in macronutrient intakes can be seen in the reported dietary choices of the UR who consumed greater amounts of high protein foods and lower amounts of foods rich in fat and sugar. It is important to account for misreporting when studying diet/disease associations in adolescents because of this bias in the reporting of macronutrient intakes.

An advantage of this work is the ability to explore differences between UR and PR in their recorded intakes of various food groups. Rather than reporting universally lower intakes than PR, UR in both sexes reported greater intakes of most core foods with the exception of dairy products, and in boys the intake of fruit, but lower intakes of sweet noncore food groups such as “biscuits, cakes and buns”, “pudding and ice cream”, and “chocolate confectionery.” Other studies have similar findings; Ventura et al. found that underreporting girls (aged 11) reported fewer servings of energy-dense foods such as pastries, French fries, and desserts, but similar numbers of servings of vegetables, fruit, and meat as PR. A study of French adolescents found that the reported intakes of pastries and cakes, ice cream, chocolate, sugar and confectionery, and sweetened beverages were lower in UR than PR. It is not possible to determine if the intake of these sweet snack foods was misremembered or deliberately excluded from the diet during the recording period.

There are several limitations to the present study. It was conducted in one geographical area of the UK in mid 2000s, which may limit its generalisability; however the cohort was reasonably representative of the UK population at recruitment, and the dietary intakes in the study were comparable with those of a nationally representative crosssectional sample NDNS (12). The assessment to classify reporting status was limited because there was no contemporaneous measure of physical activity at 13 years; thus a moderate physical activity level was assumed, and this is likely to have led to bias. Another limitation is that only 3 days of diet were recorded for most participants, 7 days of recording would have provided a more reliable estimate of intake, but this would be more burdensome on the participants and could have adversely impacted response rates and reporting accuracy. One of the main strengths of the study is its large sample size and high participation rate, although there was attrition among certain demographic groups which may have led to bias in the assessment of diet quality.

## Conclusion

In conclusion, this study shows that underreporting of dietary energy intake is very likely when asking 13-year-olds to record their food and drink consumption. This is highly biassed by body weight status and body image and has a differential effect on estimates of particular food and macronutrient intakes. Therefore, it is important to include an assessment of misreporting status when interpreting dietary information collected from young people. The study also highlights the large mismatch in girls between perception of body shape and measured levels of overweight/obesity with inappropriately high levels of dieting behaviour.

## Data Availability Statement

Publicly available datasets were analysed in this study. The study website contains details of all the data that is available through a fully searchable data dictionary and variable search tool http://www.bristol.ac.uk/alspac/researchers/our-data/.

## Ethics Statement

The studies involving human participants were reviewed and approved by the ALSPAC Ethics and Law Committee and the Local Research Ethics Committees. Written informed consent to participate in this study was provided by the participants' legal guardian/next of kin.

## Author Contributions

Data collection was carried out by the ALSPAC Study Team as part of the prospective cohort study. PE led the dietary data collection. LJ and PE conceived and designed the present study, wrote the manuscript, and act as guarantors for the integrity of the data. LJ carried out data analysis with assistance from PE. AN critically revised the manuscript. All authors have read and approved the final version.

## Funding

The UK Medical Research Council and Wellcome (Grant Ref: 217065/Z/19/Z) and the University of Bristol provide core support for ALSPAC. A comprehensive list of grants funding is available on the ALSPAC website (http://www.bristol.ac.uk/alspac/external/documents/grant-acknowledgements.pdf).

## Acknowledgement

We are extremely grateful to all the families who took part in this study, the midwives for their help in recruiting them, and the whole ALSPAC team, which includes interviewers, computer and laboratory technicians, clerical workers, research scientists, volunteers, managers, receptionists, and nurses.

## Conflict of Interest

The authors declare that the research was conducted in the absence of any commercial or financial relationships that could be construed as a potential conflict of interest.

## Publisher's Note

All claims expressed in this article are solely those of the authors and do not necessarily represent those of their affiliated organizations, or those of the publisher, the editors and the reviewers. Any product that may be evaluated in this article, or claim that may be made by its manufacturer, is not guaranteed or endorsed by the publisher.
